# Difficulties detaching psychologically from work among German teachers: prevalence, risk factors and health outcomes within a cross-sectional and national representative employee survey

**DOI:** 10.1186/s12889-021-12118-4

**Published:** 2021-11-09

**Authors:** Yasemin Z. Varol, Gerald M. Weiher, Johannes Wendsche, Andrea Lohmann-Haislah

**Affiliations:** 1 Department of Educational Psychology, Institute of Psychology, Goethe University Frankfurt, Theodor-W.-Adorno-Platz 6, D-60629 Frankfurt am Main, Germany; 2 Department of Work and Health, Federal Institute for Occupational Safety and Health, Dresden, Germany; 3 Department of Work and Health, Federal Institute for Occupational Safety and Health, Berlin, Germany

**Keywords:** Psychological detachment, Health, Recovery, Representative survey, Sickness absence, Stress, Teacher, Work

## Abstract

**Background:**

Teachers often face high job demands that might elicit strong stress responses. This can increase risks of adverse strain outcomes such as mental and physical health impairment. Psychological detachment has been suggested as a recovery experience that counteracts the stressor-strain relationship. However, psychological detachment is often difficult when job demands are high. The aims of this study were, first, to gain information on the prevalence of difficulties detaching from work among German teachers, second, to identify potential person-related/individual (i.e., age, sex), occupational (e.g., tenure, leadership position), and work-related (e.g., overload, cognitive, emotional, and physical demands) risk factors and, third, to examine relationships with mental and physical health impairment and sickness absence.

**Methods:**

A secondary analysis of cross-sectional data from a national and representative survey of German employees was conducted (BIBB/BAuA Employment Survey 2018). For the analyses data from two groups of teachers (primary/secondary school teachers: *n* = 901, other teachers: *n* = 641) were used and compared with prevalence estimates of employees from other occupations (*n* = 16,266).

**Results:**

Primary/secondary school teachers (41.5%) and other teachers (30.3%) reported more difficulties detaching from work than employees from other occupations (21.3%). Emotional demands and deadline/performance pressure were the most severe risk factors in both groups of teachers. In the group of primary/secondary school teachers multitasking demands were further risk factors for difficulties to detach from work whereas support from colleagues reduced risks. In both groups of teachers detachment difficulties can be linked to an increase in psychosomatic and musculoskeletal complaints and, additionally, to a higher risk of sickness absence among primary/secondary school teachers.

**Conclusions:**

Difficulties detaching from work are highly prevalent among German teachers. In order to protect them from related risks of health impairment, interventions are needed which aim at optimizing job demands and contextual resources (i.e., work-directed approaches) or at improving coping strategies (i.e., person-directed approaches).

## Background

The importance of teachers’ work cannot be overrated since they “are the most important in-school factor contributing to student success, satisfaction and achievement” p7 [1–3]. However, teachers face various job demands such as student misconduct, difficult interactions with colleagues or growing administrative demands [1, 4, 5]. Dealing with these demands is likely to increase teachers’ stress, which is associated with higher levels of burnout, resulting in low physical and psychological well-being [6, 7]. Studies have shown that these health problems can increase risks of higher absenteeism, early retirement and turnover intentions, leading to a teacher shortage [8–10] which is a worldwide challenge [11]. It is obvious that teachers’ stress experience might affect educational systems in total and therefore is of high importance [1, 10]. The causes for the teachers’ health impairment are diverse [12]. However “inadequate *recovery* during the teaching day” [9] p348, is named to be one of the most influential factors on teachers’ psychological stress experience [9]. Recovery is defined as the process of unwinding and restoration in which the strain level caused by work-related stressors and demands, is reduced [13]. It is an important resource to sustain well-being and mental health [13, 14]. Studies from recovery research highlight that teachers are one of the most frequently affected occupational groups suffering from recovery impairments, for instance, by difficulties in switching off mentally from job demands [15]. “Switching off” from work, also known as “psychological detachment”, describes the recovery experience of being mentally distanced from work-related thoughts during nonwork time and is suggested as one strong recovery indicator linking work stressors and strain outcomes [13, 14, 16].

The role of psychological detachment in the relationship between job demands and stress reactions is conceptualized in the ‘stressor-detachment model’ (SDM) by Sonnentag and Fritz [16]. In its basic form, the model proposes that high job demands relate to low psychological detachment and low detachment to lower well-being. Thus, this model is helpful in explaining how high job demands lead to increased stress reactions and impaired well-being by increasing risks of reduced mental recovery. These connections have been highlighted in a conceptual framework entitled recovery paradox [14]: When job demands are high and recovery is most needed, job demands reduce the ability and/or chances to fully recover from work-related stress. More specifically, one could think of teachers’ after-school activities such as correcting exams, preparing lessons for the next day at home or engaging in communication with colleagues, students and parents, all making detaching psychologically difficult, even though after dealing with such job demands this would be required. So far, meta-analytic findings with data from various occupations support the assumptions of the SDM. Psychological detachment from work negatively relates to work-related stressors and positively relates to indicators of well-being and physical and mental health [17–20], see also [21] for a summary). However, a recent review found that only a few studies investigated psychological detachment among teachers and called for more fine-grained study approaches in this profession [22]. First, it is necessary to consider work stressors that are mainly relevant to the teaching profession and to analyse them systematically. For instance, teachers’ emotional demands (e.g., difficult interactions with students or parents) or physical demands (e.g., noise) were less often considered in prior studies. Second, differentiating between different groups of teachers is important since the work of teachers and the stress level might not be comparable [22]. Contrary to that notion, van Droogenbroeck and Spruyt [23] did not find evidence for more psychological and somatic complaints in comparison to 31 other occupations and between different types of teachers. However, Stansfeld et al. [24] showed that primary and secondary school teachers report mental disorders (e.g., anxiety disorders, depression) more often than employees from other occupations.

Given this background and mixed evidence, the present study contributes to the literature in four ways. First, what we know from previous literature is that research on emotional exhaustion among teachers has focused on its relations with teacher-related well-being outcomes [25] and student outcomes [26]. Using a large and representative teacher sample allows the identification of a specific well-being indicator such as low psychological detachment that increase the risk for developing emotional exhaustion in teachers. Investigating the reasons for and consequences of low psychological detachment might allow taking preventive measures even before the health status of teachers decreases and affects student outcomes. Second, the prevalence of psychological detachment is oftentimes investigated such that teachers were frequently subsumed under the “social, cultural and education sector” [27]. As a primary focus, the following study contributes to the literature in presenting prevalence estimates of difficulties in detaching psychologically from work among German teachers. Furthermore, the prevalence rate among primary and secondary teachers is compared to other occupations as well as other types of teachers as reference groups, by using data from a large-scale national and representative survey of German employees. This goes beyond previous studies e.g., [21, 22] and allows conclusions whether primary and secondary teachers might especially be vulnerable for difficulties to psychologically detach from work. Third, we aim to identify specific risk factors of detachment difficulties in German teachers. Following the cross-occupational meta-analysis of Steed and colleagues [20], different work factors relate to psychological detachment to varying degrees. For instance, the strongest negative correlations were found for overload demands (ρ = −.30; e.g., working fast) and emotional demands (ρ = −.28; e.g., from client contacts) whereas relationships with cognitive demands (ρ = −.18; e.g., multitasking), contextual resources in the work domain (ρ = .08; e.g., colleague support), and physical demands (ρ = −.06; e.g., working under noise) are often lower on average. In the present study many personal (i.e., age, sex), occupational (e.g., tenure, employment contract, working hours, working on weekends, telework), and work-related factors that have been studied in a more piecewise fashion in earlier studies and, most importantly, less often in teachers [20, 22], are tested. The forth contribution of our study is that we investigate how difficulties detaching psychologically from work relate to indicators of teachers’ health impairment. This goes beyond existing literature by using specific rather than general well-being indicators in the context of psychological detachment [21]. More specifically, we consider indicators of mental health (i.e. sleep disturbances, exhaustion, psychosomatic complaints) and physical health (i.e. musculoskeletal complaints), and, as far as we know, for the first time sickness absence (i.e. annual days of sick leaves per year) as important health outcomes. A differentiation into specific indicators allows getting an overview of which indicators are more risky in terms of teachers’ psychological detachment. This overview also considers indicators that have so far usually been ignored (e.g. physical health indicators). The identification of most relevant indicators contributes to the literature, as this would allow the development of tailor-made interventions for teachers considering their specific work conditions.

Thus, questions that are going to be answered by the present study are: (1) What is the prevalence of difficulties detaching psychologically from work  among German teachers? (2) What are risk factors for low psychological detachment among German teachers? (3) Do difficulties detaching psychologically from work increase risks for health outcomes in German teachers?

## Methods

### Study design and data collection

The study is a secondary analysis of data from the 2018 BIBB/BAuA Employment Survey. It is the seventh national survey of German employees to describe the continuous changes in the working world. It includes questions regarding work characteristics as well as questions concerning the qualification and labour market requirements. The survey has a cross-sectional design, thus, data from waves cannot be linked to each other.

A dual frame approach was applied, meaning that not only landline but also cell phone numbers were used to gather a representative sample of German employees who were at least 15 years old and worked ten hours or more per week in a (paid) contract (i.e., exclusion of trainees and persons undergoing vocational training). The Federal Institute for Occupational Safety and Health (Germany) entrusted a social research company (Kantar Public) with data acquisition via fully standardized telephone interviews (10/2017–04/2018). From the total sample consisting of 20,012 participants from different occupations, we considered 17,828 employees for our (unweighted) analyses (i.e., the self-employed, freelancers and family workers were excluded). Rohrbach-Schmidt and Hall [28] give a full report on sampling, data collected, and methods used in this survey.

This paper specifically concerns the population of German teachers (*n* = 1547) and compares prevalence estimates of difficulties in detaching psychologically from work during nonwork time with the remaining employees from other occupations (*n* = 16,281). We identified occupational groups according to the KLDB 2010 (‚Klassifikation der Berufe’ ~ [German] classification of occupations, similar to the international standard classification of occupations ISCO).

The comparison with “other occupations” follows the Stress Report 2019, which shows that employees from the education sector differ in their psychological detachment level in comparison to other occupations [27]. In our study, we stratified analyses for two groups of teachers, since both differ with regard to work tasks, type of students, and work characteristics. The first group consists of primary and secondary school teachers (*n* = 906). The second group is composed of other teachers (*n* = 641) who work at specialized schools with disabled children, at vocational schools with primarily older students and adults, at universities, in adult education (i.e., training for professional development), and in institutions of non-formal education (e.g., music teachers, art teachers, and sports teachers). A further investigation of teacher subgroups was not possible, as only small sub-sample sizes occurred here. For such analyses, other studies are needed that would have to target teachers only. Working conditions might be very diverse here. However, these differences are controlled for in our analyses or are themselves the subject of the analysis. We conducted all analyses regarding risk factors as well as health outcomes in relation to detachment difficulties only for the target population of teachers.

### Variables and measures

#### Difficulties detaching psychologically from work during nonwork time

*Difficulties detaching from work* was assessed by a single item ‘How often do you find it difficult to detach from work during nonwork time?’. The response format was a four-point-frequency scale (1 = ‘never’, 2 = ‘seldom’, 3 = ‘sometimes’, 4 = ‘often’). For the analyses on predictors and outcomes of low psychological detachment, responses were categorized to 0 = low difficulties (‘never, seldom, sometimes’) and 1 = strong difficulties (‘often’). The response format is equal to the format of the predictor variables scales.

#### Demographic predictors

Demographic variables included *sex* (1 = ‘male’, 2 = ‘female’) and *age* (1 = ‘< 37 years’, 2 = ‘37–50 years’, 3 = ‘> 50 years’).

#### Occupational predictors

*Organizational tenure* (1 = ‘less than 5 years’, 2 = ‘5–14 years’, 3 = ‘more than 14 years’) and *job tenure* (1 = ‘less than 4 years’, 2 = ‘4–10 years’, 3 = ‘more than 10 years’), *leadership position* (0 = ‘no’, 1 = ‘yes’), *employment contract* (0 = ‘permanent’, 1 = ‘temporary’), actual *weekly working hours* (1 = ‘< 21 hours/week, 2 = ‘21-34 hours/week’, 3 = ‘35-41 hours/week’, 4 = ‘> 41 hours/week’), *working on weekends* (0 = ‘no’, 1 = ‘yes’), and *telework* (0 = ‘no’, 1 = ‘yes’) were assessed as occupational predictors.

#### Overload demands as predictors

Overload demands at work were assessed with three items on *deadline and performance pressure* (‘How often in your work does it occur that you have to work under strong deadline or performance pressure?’), *working very fast* (i.e., time pressure; ‘How often in your work does it occur that you have to work very fast?’), and *prescribed performance standards* (‘How often in your work does it occur that you are prescribed an exact number of pieces, a certain minimum output or the time to do a certain task?’). Responses were categorized as 0 = low demands (‘never, seldom, sometimes’) and 1 = high demands (‘often’).

#### Cognitive demands as predictors

We assessed cognitive demands at work with two items on *multitasking* (‘How often in your work does it occur that you have to keep an eye on different types of work or processes at the same time?’), and *work interruptions* (‘How often in your work does it occur that you get disturbed or interrupted at work, e.g. by colleagues, insufficient working materials, machine malfunctions or telephone calls?’). Responses were categorized as 0 = low demands (‘never, seldom, sometimes’) and 1 = high demands (‘often’).

#### Emotional demands as predictors

We assessed emotional demands at work with the item ‘How often in your work does it occur that your job puts you in situations that cause you to experience emotional stress?’. Responses were categorized as 0 = low demands (‘never, seldom, sometimes’) and 1 = high demands (‘often’).

#### Contextual resources as predictors

We assessed contextual resources at work with three items on *colleague support* (‘How often do you get help and support for your work from colleagues when you need it?’), *supervisor support* (‘How often do you get help and support for your work from your direct supervisor when you need it?’), and *rewards from supervisor* (‘How often does your direct supervisor express praise and recognition for your work?’). Responses were categorized as 0 = low resources (‘never, seldom, sometimes’) and 1 = high resources (‘often’).

#### Physical demands as predictors

We assessed noise as typical physical demand for the work of teachers (‘How often do you have to work in a noisy environment?’ and ‘How often do you have to work in a distractingly noisy environment?’). Responses were categorized as 0 = low demands (‘never, seldom, sometimes’) and 1 = high demands (‘often’).

#### Health-related outcomes

During the survey, eight *psychosomatic complaints* were assessed with a checklist (responses 0 = ‘no’, 1 = ‘yes’) in response to the item ‘Please tell me if the following complaints have occurred in the last 12 months during your work or on working days’. Complaints were general fatigue/tiredness/exhaustion, headache, stomach/digestive disorders, nervousness/irritability, sleep disorders at night, dejectedness, physical exhaustion, and emotional exhaustion. After calculating a sum score indicating symptom severity, responses were categorized as 0 = ‘no symptoms’, 1 = ‘1 symptom, and 2 = ‘more than 1 symptom’.

Moreover, detailed analyses were conducted for *sleep disturbances* (response to item ‘sleep disorders at night) as outcome and *exhaustion* as outcome (sum score for responses to the items ‘general fatigue/tiredness/exhaustion’, ‘physical exhaustion’, and ‘emotional exhaustion’; 0 = ‘no symptoms’, 1 = ‘1 symptom, and 2 = ‘more than 1 symptom’).

Furthermore, *musculoskeletal complaints* were assessed with a similar checklist of eight physical symptoms (‘Please tell me if the following complaints have occurred in the last 12 months during your work or on working days’: lower back pain/low back pain responses, pain in the neck/shoulder area, pain in arms, pain in hands, hip pain, pain in the knees, swollen legs, pain in the legs/feet) with responses ranging from 0 = ‘no’ to 1 = ‘yes’. After calculating a sum score indicating symptom severity, responses were categorized as 0 = ‘no symptoms’, 1 = ‘1 symptom, and 2 = ‘more than 1 symptom’.

*Sickness absence* was assessed with the item ‘How many days have you stayed ill at home during the last 12 months’. Responses were categorized as 0 = ‘no days’, 1 = ‘1–5 days’, and 2 = ‘more than 5 days’.

#### Data processing and statistical analysis

Complete data was available for 15,813 persons (88.7%). Missing data was not at random (Little’s MCAR-Test: χ^2^ = 7493.71, *p* < .001) and highest for job tenure (with 2.8%). For analyses regarding risk factors of detachment difficulties and related health outcomes, we applied multiple data imputation of missing data (*k =* 5; cf.) [29] and report pooled results among datasets.

Considering information on prevalence, frequencies of responses and corresponding 95% confidence intervals were calculated for all four possible responses in relation to the item on difficulties to detach psychologically from work. Unweighted (raw) and weighted results are reported. Rohrbach and Schmidt [28] describe the weighting algorithm relating to an adjustment of data according to official data from the 2017 micro census. The estimates relating to the two subsamples of teachers are compared with the sample of employees from other occupations.

Odds ratios (OR) and 95% confidence intervals (95% CI) were calculated to examine the relationships of difficulties detaching psychologically from work with demographic, occupational, and work-related variables (overload demands, cognitive demands, emotional demands, contextual resources, and physical demands) using binary logistic regression. First univariate analyses were conducted and psychological detachment difficulties as outcome were regressed on each predicting variable as independent variable. In a second step, a multivariate logistic regression analysis was conducted, integrating all predictors into the model in parallel.

Moreover, ORs and corresponding 95% CIs for difficulties detaching psychologically from work in relation to the five health-related outcomes (sleep disturbances, exhaustion, psychosomatic complaints, musculoskeletal complaints, and sickness absence) were calculated. In a first step, univariate logistic regression analyses were conducted. We compared these results with estimates from additional multivariate logistic regression analyses that were adjusted for demographic, occupational, and work-related variables (see above). We considered parameter estimates with *p*-values lower .05 as significant. All analyses were conducted using IBM SPSS Statistics 26.0.

## Results

Table 1 contains descriptive information on distributions of person-related, work-related, and health-related variables among German primary/secondary school teachers and other teachers (e.g., teachers who work at specialized schools with disabled children or at vocational schools with primarily older students and adults as described in the method section). We found many differences between both subsamples of teachers.

**Table 1:** Person-related, Work-related, and Health-related Variables of German Primary/Secondary School Teachers (*n* = 906) and Other Teachers (*n* = 641)

		Primary/Secondary school teachers		Other teachers		
		n	%	n	%	*p*
Person-related variables						
*Demographic variables*						
Sex	Male	251	27.7	314	49.0	**<.001**
	Female	655	72.3	327	51.0	
Age	< 37 years	165	18.4	165	25.9	**.002**
	37–50 years	308	34.3	198	31.0	
	> 50 years	426	47.4	275	43.1	
Work-related variables						
*Occupational variables*						
Organizational tenure	< 5 years	151	16.8	189	29.5	**<.001**
	5–14 years	276	30.7	217	33.9	
	> 14 years	473	52.6	234	36.6	
Job tenure	< 4 years	190	21.2	210	33.1	**<.001**
	4–10 years	266	29.6	192	30.2	
	> 10 years	442	49.2	233	36.7	
Leadership position	No	732	80.9	430	67.2	**<.001**
	Yes	173	19.1	210	32.8	
Employment contract	Permanent	869	95.9	463	72.2	**<.001**
	Temporary	37	4.1	178	27.8	
Weekly working hours	< 21 h	82	9.1	81	12.6	**.015**
	21–34 h	170	18.8	89	13.9	
	35–41 h	309	34.1	232	36.2	
	> 41 h	345	38.1	239	37.3	
Working on weekends	No	479	53.0	376	58.9	**.021**
	Yes	425	47.0	262	41.1	
Telework	No	100	11.1	114	17.8	**<.001**
	Yes	804	88.9	525	82.2	
*Overload demands*						
Deadline/Performance pressure	low	375	41.4	345	53.9	**<.001**
	high	531	58.6	295	46.1	
Working very fast	low	695	77.1	524	82.0	**.021**
	high	206	22.9	115	18.0	
Prescribed performance standards	low	628	69.6	493	77.2	**.001**
	high	274	30.4	146	22.8	
*Cognitive demands*						
Multitasking	low	149	16.4	207	32.3	**<.001**
	high	757	83.6	434	67.7	
Work interruptions	low	504	55.7	413	64.4	**<.001**
	high	401	44.3	228	35.6	
*Emotional demands*						
Emotional demands	low	631	69.6	563	87.8	**<.001**
	high	275	30.4	78	12.2	
*Contextual resources*						
Colleague support	low	164	18.2	128	20.0	.371
	high	739	81.8	513	80.0	
Supervisor support	low	390	43.7	266	42.8	.746
	high	503	56.3	355	57.2	
Rewards (Supervisor)	low	624	69.6	419	67.7	.439
	high	273	30.4	200	32.3	
*Physical demands*						
Noise	low	416	46.0	565	88.1	**<.001**
	high	489	54.0	76	11.9	
Distracting noise	low	548	60.6	559	87.2	**<.001**
	high	357	39.4	82	12.8	
Health-related variables						
*Mental health*						
Sleep disturbances	No	558	61.7	460	71.8	**<.001**
	Yes	346	38.3	181	28.2	
Exhaustion	No	293	32.5	304	47.4	**<.001**
	1 symptom	161	17.9	103	16.1	
	> 1 symptom	447	49.6	234	36.5	
Psychosomatic complaints	No	182	20.3	227	35.6	**<.001**
	1 symptom	130	14.5	101	15.8	
	> 1 symptom	585	65.2	310	48.6	
*Physical health*						
Musculoskeletal complaints	No	301	33.3	248	38.8	.065
	1 symptom	237	26.2	145	22.7	
	> 1 symptom	366	40.5	246	38.5	
*Sickness absence*						
Annual sick leave (days)	No	286	31.8	228	35.7	.130
	1–5 days/year	317	35.3	196	30.7	
	> 5 days/year	295	32.9	214	33.5	

*Note. p*-values of Χ^2^-test. Values lower .05 are boldfaced. Sums of cell *n*s might not be equal to total *n*s because of missing data

Regarding *person-related variables* primary/secondary school teachers were more often female and older than the other teachers were.

In relation to *work-related variables*, primary/secondary school teachers more often reported higher organizational tenure and job tenure, a permanent employment contract, longer weekly working hours, working on weekends, and telework. The group of other teachers more often reported a leadership position than primary/secondary school teachers. In addition, primary/secondary school teachers more often reported overload demands, cognitive demands, emotional demands, and physical demands than other teachers. There were no significant differences between both groups of teachers in relation to contextual resources such as colleague and supervisor support and rewards.

Considering *health-related variables*, primary/secondary school teachers more often reported sleep disturbances, exhaustion, and psychosomatic complaints than other teachers. There were no significant differences in relation to musculoskeletal complaints and annual sick leave.

### Prevalence of problems detaching psychologically from work

Table 2 shows prevalence estimates of difficulties detaching psychologically from work among German teachers and among employees from other occupations.

**Table 2:** Unweighted and Weighted Prevalence Estimates for Responses of Difficulties Detaching from Work among German Teachers and among Employees from Other Occupations

Responses	Primary/Secondary school teachers	Other teachers^a^	Other occupations
Total *N*	906	640	16,266
Never			
n	60	96	4194
% (Unweighted)	6.6	15.0	25.8
95% CI (Unweighted)	[5.1, 8.4]	[12.4, 17.9]	[25.1, 26.5]
% (Weighted)	6.7	20.9	29.0
95% CI (Weighted)	[5.0, 8.9]	[16.1, 26.7]	[28.0, 30.0]
Rarely			
n	163	144	4080
% (Unweighted)	18.0	22.5	25.1
95% CI (Unweighted)	[15.6, 20.6]	[19.4, 25.9]	[24.4, 25.8]
% (Weighted)	18.8	23.1	24.3
95% CI (Weighted)	[15.8, 22.3]	[18.6, 28.3]	[23.4, 25.2]
Sometimes			
n	307	206	4524
% (Unweighted)	33.9	32.2	27.8
95% CI (Unweighted)	[30.9, 37.0]	[28.7, 35.9]	[27.1, 28.5]
% (Weighted)	33.7	27.2	25.9
95% CI (Weighted)	[29.8, 37.8]	[22.5, 32.4]	[25.0, 26.9]
Often			
n	376	194	3468
% (Unweighted)	41.5	30.3	21.3
95% CI (Unweighted)	[38.3, 44.7]	[26.8, 34.0]	[20.7, 22.0]
% (Weighted)	40.7	28.9	20.8
95% CI (Weighted)	[36.5, 45.1]	[23.9, 34.4]	[19.9, 21.7]

*Note.* Raw data was used. 95% CI (confidence interval) with lower and upper limits in brackets. Unweighted ns reported

^a^ Estimates from data set with one missing

Responses towards difficulties detaching psychologically from work significantly differed between both groups of teachers and employees from other occupations (χ^2^ (6, *N* = 17,812) = 221.48, *p <* .001). Considering the unweighted and weighted prevalence estimates, primary and secondary school teachers more often reported difficulties detaching psychologically from work (41.5and 40.7%) than other teachers (30.3 and 28.9%; χ^2^ (1, *N* = 1546) = 20.17, *p <* .001) and more often problems than employees from other occupations (21.3 and 20.8%; χ^2^ (1, *N* = 17,172) = 201.16, *p <*. 001). Moreover, the group of other teachers more often reported difficulties detaching psychologically from work than employees from other occupations (χ^2^ (1, *N* = 16,906) = 29.34, *p <.* 001).

In summary, German teachers, especially those working in primary and secondary schools, report difficulties detaching psychologically from work more often in comparison to employees from other occupations.

### Person-related, occupational and work-related risk factors

Table 3 shows results from logistic regression analyses regarding the potential risk factors in relation to difficulties detaching from work.

**Table 3:** Anteceding Variables of Reporting Difficulties Detaching from Work among German Teachers

	Primary/Secondary school teachers (n = 906)								Other teachers (n = 641)							
	Detachment difficulties		Univariate model			Multivariate model			Detachment difficulties		Univariate model			Multivariate model		
	Low (%)	High (%)	OR	LL	UL	OR	LL	UL	Low (%)	High (%)	OR	LL	UL	OR	LL	UL
*Demographic variables*																
Sex																
Male	63.3	36.7	1			1			73.2	26.8	1			1		
Female	56.6	43.4	1.32	0.98	1.79	1.28	0.90	1.82	66.4	33.6	1.39	0.99	1.95	1.08	0.72	1.62
Age																
< 37 years	56.0	44.0	1			1			74.0	26.0	1			1		
37–50 years	63.4	36.6	0.74	0.50	1.08	0.74	0.45	1.23	71.3	28.7	1.15	0.72	1.82	0.93	0.52	1.67
> 50 years	55.8	44.2	1.01	0.70	1.45	0.97	0.55	1.70	66.0	34.0	1.46	0.95	2.25	1.16	0.60	2.24
*Occupational variables*																
Organizational tenure																
< 5 years	59.6	40.4	1			1			68.8	31.2	1			1		
5–14 years	63.5	36.5	0.85	0.57	1.28	0.73	0.38	1.40	74.2	25.8	0.77	0.50	1.18	**0.46**	**0.23**	**0.94**
> 14 years	55.2	44.8	1.19	0.82	1.73	0.97	0.48	1.97	66.3	33.7	1.12	0.74	1.68	0.67	0.28	1.60
Job tenure																
< 4 years	60.5	39.5	1			1			71.7	28.3	1			1		
4–10 years	57.6	42.4	1.13	0.77	1.65	1.28	0.72	2.26	68.8	31.2	1.14	0.75	1.76	1.72	0.87	3.40
> 10 years	58.1	41.9	1.10	0.78	1.56	0.93	0.51	1.72	68.7	31.3	1.15	0.76	1.73	1.25	0.54	2.86
Leadership position																
No	57.8	42.2	1			1			70.1	29.9	1			1		
Yes	61.4	38.6	0.86	0.61	1.21	0.81	0.54	1.22	69.0	31.0	1.05	0.74	1.51	0.78	0.50	1.22
Employment contract																
Permanent	58.1	41.9	1			1			68.9	31.1	1			1		
Temporary	67.6	32.4	0.67	0.33	1.34	0.85	0.36	2.01	71.9	28.1	0.87	0.59	1.27	1.07	0.61	1.88
Weekly working hours																
< 21 h	69.5	30.5	1			1			76.5	23.5	1			1		
21–34 h	64.7	35.3	1.24	0.71	2.19	1.16	0.61	2.20	68.5	31.5	1.50	0.76	2.96	1.64	0.76	3.56
35–41 h	61.8	38.2	1.41	0.83	2.38	1.15	0.62	2.11	72.8	27.2	1.22	0.67	2.19	1.25	0.63	2.48
> 41 h	49.9	50.1	**2.29**	**1.37**	**3.84**	1.66	0.89	3.11	64.8	35.2	1.77	0.99	3.15	1.44	0.70	2.96
Working on weekends																
No	63.4	36.6	1			1			73.7	26.3	1			1		
Yes	53.0	47.0	**1.54**	**1.18**	**2.01**	1.31	0.94	1.81	64.0	36.0	**1.58**	**1.13**	**2.23**	1.40	0.93	2.10
Telework																
No	67.9	32.1	1			1			73.7	26.3	1			1		
Yes	57.3	42.7	**1.58**	**1.01**	**2.46**	1.02	0.60	1.75	68.9	31.1	1.27	0.80	2.01	1.05	0.61	1.80
*Overload demands*																
Deadline/Performance pressure																
Low	72.8	27.2	1			1			79.1	20.9	1			1		
High	48.4	51.6	**2.85**	**2.15**	**3.79**	**1.97**	**1.42**	**2.75**	58.8	41.2	**2.65**	**1.87**	**3.75**	**1.63**	**1.05**	**2.52**
Working very fast																
Low	60.7	39.3	1			1			73.7	26.3	1			1		
High	51.3	48.7	**1.47**	**1.07**	**2.00**	0.81	0.55	1.19	51.7	48.3	**2.62**	**1.73**	**3.96**	1.52	0.92	2.49
Prescribed performance standards																
Low	61.9	38.1	1			1			73.0	27.0	1			1		
High	50.7	49.3	**1.58**	**1.19**	**2.10**	1.23	0.88	1.73	58.9	41.1	**1.88**	**1.28**	**2.77**	1.35	0.86	2.13
*Cognitive demands*																
Multitasking																
Low	76.5	23.5	1			1			75.8	24.2	1			1		
High	55.0	45.0	**2.67**	**1.78**	**4.00**	**1.67**	**1.05**	**2.66**	66.8	33.2	**1.56**	**1.07**	**2.27**	0.97	0.62	1.51
Work interruptions																
Low	64.8	35.2	1			1			74.1	25.9	1			1		
High	50.6	49.4	**1.79**	**1.37**	**2.34**	1.06	0.76	1.48	61.8	38.2	**1.76**	**1.25**	**2.49**	1.39	0.91	2.12
*Emotional demands*																
Emotional demands																
Low	71.6	28.4	1			1			75.7	24.3	1			1		
High	28.4	71.6	**6.38**	**4.66**	**8.73**	**5.55**	**3.96**	**7.78**	26.9	73.1	**8.44**	**4.94**	**14.43**	**5.84**	**3.25**	**10.46**
*Contextual resources*																
Colleague support																
Low	49.2	50.8	1			1			58.6	41.4	1			1		
High	60.6	39.4	**0.63**	**0.45**	**0.88**	**0.63**	**0.42**	**0.96**	72.5	27.5	**0.54**	**0.36**	**0.80**	0.82	0.50	1.34
Supervisor support																
Low	52.8	47.2	1			1			63.1	36.9	1			1		
High	62.9	37.1	**0.66**	**0.50**	**0.87**	0.93	0.65	1.32	74.7	25.3	**0.58**	**0.41**	**0.82**	0.72	0.46	1.13
Rewards (Supervisor)																
Low	56.8	43.2	1			1			68.2	31.8	1			1		
High	62.2	37.8	0.80	0.59	1.07	0.91	0.63	1.30	72.9	27.1	0.80	0.55	1.16	1.16	0.72	1.85
*Physical demands*																
Noise																
Low	65.1	34.9	1			1			71.7	28.3	1			1		
High	52.9	47.1	**1.66**	**1.27**	**2.17**	1.07	0.75	1.53	55.3	44.7	**2.05**	**1.26**	**3.34**	1.16	0.61	2.21
Distracting noise																
Low	63.6	36.4	1			1			71.7	28.3	1			1		
High	50.6	49.4	**1.71**	**1.30**	**2.24**	1.14	0.80	1.64	56.1	43.9	**1.99**	**1.24**	**3.19**	1.14	0.62	2.09

*Note.* Imputed data set was used for analyses. LL/UL = lower and upper limit of ORs 95% confidence interval. ‘low’ = never, rarely, sometimes, ‘high’ = often. R^2^ in the multivariate model was .29 for primary/secondary school teachers and .23 for other teachers. ORs and corresponding 95% confidence intervals in boldface are significant with *p* < .05

Results from the univariate analyses were similar across both groups of teachers. Teachers reporting increased weekly working hours, working on weekends, telework (not significant in the sample of other teachers), increased overload demands (i.e., deadline/performance pressure, working very fast, prescribed performance standards), increased cognitive demands (i.e., multitasking, work interruptions), increased emotional demands, less contextual resources (i.e., colleague and supervisor support), and increased physical demands (i.e., noisy work environment) were at higher risk to report difficulties detaching psychologically from work during nonwork time.

However, when considering all these risk factors in combination (multivariate models), we found the following significant and substantial risk factors for difficulties detaching psychologically among primary/secondary school teachers and other teachers: more often reporting emotional demands (ORs: 5.55 and 5.84) and more often reporting deadline/performance pressure (ORs: 1.97 and 1.63). For primary/secondary school teachers multitasking demands were a further risk factor (OR = 1.67) and, in addition, experiencing colleague support more often was associated with reduced risks (OR = 0.63).

### Health-related outcomes

Finally, we examined if difficulties with psychological detachment increase risks for health impairment. Results from the multivariate regression analyses (adjusting for other person-related, occupational and work-related variables; see Table 4) widely supported this assumption. Primary/secondary school teachers and other teachers who reported difficulties with psychological detachment more often were at higher risk reporting sleep disturbances (ORs: 4.73 and 3.43), more than one symptom of exhaustion (ORs: 3.86 and 4.19), more than one psychosomatic complaint (ORs: 4.42 and 4.27), and more than one musculoskeletal complaint (ORs: 1.52 and 1.59). Moreover, we found a significantly increased risk for reporting more than five annual sick leave days (OR = 1.75) in the group of primary/secondary school teachers but not in the group of other teachers (OR = 1.43) that was, however, significant in the univariate analysis (OR = 1.86).

**Table 4:** Difficulties Detaching from Work and Health Outcomes among German Teachers

		Outcome categories			Categories 1 vs. 2		Categories 1 vs. 3	
					Univariate	Multivariate	Univariate	Multivariate
		1	2	3	*OR* (95%CI)	*OR* (95%CI)	*OR* (95%CI)	*OR* (95%CI)
Primary/Secondary school teachers (n = 906)								
*Sleep disturbances*		No (%)	Yes (%)					
Detachment difficulties	low	77.5	22.5		1	1		
	high	39.5	60.5		**5.26 (3.93, 7.04)**	**4.73 (3.37, 6.64)**		
*Exhaustion*		No (%)	1 symptom (%)	> 1 symptom (%)				
Detachment difficulties	low	44.5	19.3	36.3	1	1	1	1
	high	15.4	16.1	68.4	**2.41 (1.57, 3.70)**	**2.29 (1.40, 3.74)**	**5.44 (3.86, 7.66)**	**3.86 (2.59, 5.75)**
*Psychosomatic complaints*		No (%)	1 symptom (%)	> 1 symptom (%)				
Detachment difficulties	low	29.1	19.5	51.4	1	1	1	1
	high	7.7	7.6	84.7	1.46 (0.82, 2.60)	1.05 (0.53, 2.09)	**6.21 (4.05, 9.54)**	**4.42 (2.76, 7.08)**
*Musculoskeletal complaints*		No (%)	1 symptom (%)	> 1 symptom (%)				
Detachment difficulties	low	38.2	26.6	35.2	1	1	1	1
	high	26.3	25.8	47.9	1.40 (0.99, 2.00)	1.25 (0.83, 1.88)	**1.97 (1.44, 2.71)**	**1.52 (1.04, 2.24)**
*Annual sick leave (days)*		No (%)	1–5 days (%)	> 5 days (%)				
Detachment difficulties	low	35.9	37.1	27.0	1	1	1	1
	high	25.6	32.5	41.9	1.23 (0.88, 1.72)	1.14 (0.77, 1.68)	**2.18 (1.56, 3.04)**	**1.75 (1.19, 2.58)**
Other teachers (n = 641)								
*Sleep disturbances*		No (%)	Yes (%)					
Detachment difficulties	low	81.4	18.6		1	1		
	high	49.5	50.5		**4.48 (3.10, 6.47)**	**3.43 (2.27, 5.20)**		
*Exhaustion*		No (%)	1 symptom (%)	> 1 symptom (%)				
Detachment difficulties	low	58.6	15.7	25.7	1	1	1	1
	high	21.6	17.0	61.3	**2.94 (1.74, 4.98)**	**2.70 (1.51, 4.83)**	**6.46 (4.26, 9.77)**	**4.19 (2.57, 6.82)**
*Psychosomatic complaints*		No (%)	1 symptom (%)	> 1 symptom (%)				
Detachment difficulties	low	44.6	18.2	37.1	1	1	1	1
	high	14.4	10.3	75.2	1.75 (0.93, 3.29)	1.57 (0.77, 3.19)	**6.27 (3.98, 9.87)**	**4.27 (2.56, 7.10)**
*Musculoskeletal complaints*		No (%)	1 symptom (%)	> 1 symptom (%)				
Detachment difficulties	low	43.0	23.3	33.6	1	1	1	1
	high	28.9	21.6	49.5	1.38 (0.87, 2.20)	1.20 (0.69, 2.07)	**2.19 (1.48, 3.25)**	**1.59 (1.01, 2.51)**
*Annual sick leave (days)*		No (%)	1–5 days (%)	> 5 days (%)				
Detachment difficulties	low	38.5	31.3	30.1	1	1	1	1
	high	28.9	29.0	42.0	1.23 (0.80, 1.90)	1.37 (0.83, 2.26)	**1.86 (1.24, 2.80)**	1.43 (0.90, 2.29)

*Note.* Imputed data set was used for analyses. 95% CI = 95% confidence interval. ‘low’ = never, rarely, sometimes, ‘high’ = often. ORs and corresponding 95% CIs in boldface are significant with *p* < .05. Multivariate models are adjusted for sex, age, organizational tenure, job tenure, leadership position, employment contract, weekly working hours, working weekends, telework, deadline/performance pressure, working very fast, prescribed performance standards, multitasking, work interruptions, emotional demands, colleague support, supervisor support, rewards (supervisor), noise, and distracting noise

## D**iscussion**

### General discussion of results

The results of the present study underline that the prevalence of difficulties detaching psychologically from work during nonwork time is rather high among German teachers (research question 1). It is overall higher than in other occupations and highest among primary and secondary school teachers in particular. Psychological detachment seems to be a serious issue within the teaching profession because results from meta-analyses [18, 20] suggest this variable to be an important element of recovery process that is central for maintaining mental and physical health.

In both groups of teachers, we found rather consistent risk factors for difficulties detaching psychologically from work (research question 2). More specifically, overload demands (deadline and performance pressure) and emotional demands were significant risk factors. For primary and secondary teachers multitasking as an indicator of cognitive demands is a further risk factor. In contrast, colleague support is associated with a reduced risk for difficulties detaching psychologically from work. These findings suggest that especially finishing tasks and effectively regulating emotions go hand in hand with higher psychological detachment. In addition to these findings, occupational demands (i.e., weekly working hours, working on weekends, telework, work interruptions), supervisor support and physical demands are no significant risk factors in combination with the aforementioned variables in one analysis.

The study results also confirm the hypothesized impact of detachment difficulties for an increased risk of health impairment (research question 3). Consistent with earlier meta-analytic findings [20], we found in this study that difficulties detaching psychologically from work relates positively to mental health indicators such as exhaustion and sleep disturbance. While recent meta-analytic findings could not report any correlations with physical well-being indicators due to insufficient data [20], the present study with representative data shows that difficulties detaching psychologically is a risk factor for low physical health (e.g. musculoskeletal complaint) and sickness absence.

### Study limitations and future research

The findings of this study have to be seen in light of some limitations. First, the study is cross-sectional in nature, which does not allow further suggestions on the causality of the relationship between difficulties detaching psychologically from work, work factors, and health. What we cannot conclude based on the data is whether the relationships between risk and outcome factors of psychological detachment are unidirectional. One may argue [14] that, for example, sleep disturbance as a possible outcome also can be a risk factor for difficulties detaching psychologically, suggesting a reciprocal relationship. Second, the research was conducted with representative data of German teachers. This raises the question, whether the data is generalizable across countries and cultures. Of course, educational systems differ between countries and research showed that the average working hours differ between teachers from different countries [30]. However, working hours and occupational demands are not the most important predictors of psychological detachment in German teachers. It is therefore feasible to suggest that when emotional demands and overload demands are high teachers in other countries might as well experience lower levels of psychological detachment [30]. Third, all variables were assessed via self-reports and (nearly) all with single-item measures (health outcomes with multi-item symptom checklists), which might bias results by common-method variance [31] and low reliability. However, psychological detachment is by definition a subjective recovery experience [16] that needs individuals’ subjective statements for assessment. Therefore, self-reports are typical for this kind of research, also when considering potential antecedents and outcomes of detachment from work [18]. Using a single-item measure for detachment difficulties is also more warranted since a meta-analysis showed that items of established scales are highly homogenous (α ≥ .86; see) [32]. Moreover, using single-item-measures might be appropriate when a study’s primary foci are on content validity, concrete and unidimensional constructs, being the case here [33, 34]. An advantage of our study approach is also the large and representative population. Since the BIBB/BAuA survey covers many aspects on employees work and health, available time during interviews is limited and short assessments of constructs are required in order to reduce participant’s load and the risk of opt outs.

Related to the first limitation, the approach of the present study captures all constructs as more trait-like and stable variables although at least the measures of health-outcomes included a component of time (i.e., 12-month reference of symptoms). However, research has shown that constructs such as psychological detachment, work stressors, well-being, and health include substantial within-person variance [35, 36]. This means that they do not only vary between persons but also within persons from day to day. Although prior research showed that correlations of psychological detachment in relation to risk factors and outcomes are often stronger on the between-person than on the within-person level [37] only a few diary studies on teachers detachment have been published so far [22]. Therefore, future research should use such micro longitudinal study designs more often here, also because prior research showed that within-person variability of constructs might even moderate between-person relationships, for instance, between job demands and strain outcomes [38]. Finally, there is evidence that trait variables such as workaholism, overcommitment, or negative affectivity and neuroticism [18] as well as personal resources such as self-efficacy [20] affect psychological detachment. In the present study, these trait variables were not controlled for. However, meta-analytic evidence showed that the associations between job demands, psychological detachment, and well-being are still significant when the trait variables are considered [39]. Beyond personal characteristics, work characteristics such as quantitative and emotional demands still appear to be important, strengthening the conclusions of the results presented in this paper.

### Practical implications

From a practical point of view, our results suggest that interventions improving psychological detachment from work are urgently needed for German teachers. Such interventions might directly target work-related risk factors (i.e., work-directed interventions) or change employees’ strategies for coping with work-stressors and strain outcomes (i.e., person-directed interventions) [40];. Karabinski and colleagues [41] recently reviewed the literature on such interventions. They found that work-directed interventions aiming to reduce job demands have largely not been investigated so far and that average effect sizes are rather low (d = 0.14). In contrast, person-directed interventions produce on average a substantial improvement in psychological detachment (d = 0.39), especially those training programs that foster a beneficial reappraisal of work stressors (e.g., emotion regulation: d = 0.48; boundary management: d = 0.65; sleep: d = 0.88). Moreover, results of this meta-analysis showed that intervention effects can be strengthened when targeting to employees with ongoing recovery-related impairments (e.g., sleep disturbances) and using high-dose and spaced training approaches (i.e., more than two weeks duration, more than four hours of total training). Since intervention effects seem to decrease after six months, booster sessions might improve sustainability. Another meta-analysis [42] showed that such interventions not only reduce difficulties in detaching psychologically from stressors but also improve health behaviour (g = 0.31) and physical health (g = 0.23), thus directly improving employees’ health.

However, from a theoretical (i.e., stressor-detachment-model), [16] and a public health perspective, approaches that aim to reduce work-related risk factors are valuable as more employees would benefit from them. Theory in educational psychology emphasizes the necessity for teachers to effectively deal with the numerous demands of the teaching profession and to develop a healthy distance to school [43]. The present study highlights that not every stressor is equally related to poor recovery and health outcomes. In line with the recovery research [20], overload demands and emotional demands impact recovery of teachers more compared to qualitative and cognitive demands. According to the results, this would necessitate a reduction of teachers’ time/performance pressure, their emotional demands from interactions with students and their parents, and multitasking demands as well as increasing chances to get emotional support from their colleagues. This might lead to significant difficulties, since job-crafting interventions, in which teachers are asked to set goals to change their work environment and relationships to colleagues, showed that teachers oftentimes do not try to (or experience difficulties to) decrease job demands and increase social support. Therefore, teachers might need ideas and help that are more external to change their work environment [45–47]. Iancu et al. [48] concluded that most intervention studies aiming to reduce teachers’ exhaustion levels do not address teacher-specific stressors. However, there are some approaches and interventions to foster stress management abilities, mental distancing, or well-being in teachers. To deal with stressors within the classroom, one example are training programs, which strengthen personal abilities in classroom management in order to improve students’ academic and social–emotional learning [49] p4. Dicke et al. [50], for instance, implemented a classroom management program for student teachers and showed that it reduced their emotional exhaustion and increased their job satisfaction [51]. It might be promising to investigate further if classroom management interventions do also improve teachers’ psychological detachment. To directly target teachers’ mental recovery, Ebert and colleagues implemented a multicomponent training intervention which included recovery-related psychoeducational elements, and cognitive-behavioral techniques such as detached mindfulness or attention training [52, 53]. This intervention improved mental distancing (i.e., psychological detachment) and reduced sleep problems. Considering the work-related stressors, time or performance pressure might also indirectly affect detachment by reducing time for recovery, for instance, by skipping mandatory rest breaks. Although time for long lunch breaks might be limited for teachers at school, school principals might encourage their teachers scheduling short rest breaks, which have been shown to reduce feelings of stress and fatigue and to strengthen social support and task performance [54, 55]. Detachment during rest breaks might also lead to lower negative affect and higher positive affect in the afternoon [56]. However, not only individual level interventions might be crucial for teachers’ psychological detachment: Work conditions within the educational system should promote recovery and detaching from work. For example, it has been shown that employees who perceived their supervisors as supporting recovery, experienced higher psychological detachment [57]. Therefore, school management emphasizing the importance of recovery experience is likely to positively impact teachers’ actual recovery experience and psychological detachment.

## Conclusions

Difficulties detaching psychologically from work are highly prevalent among German teachers. This has implications for educational systems and public health. It has been shown that teachers with poor mental health and various forms of psychological disorders tend to have higher intention to quit work [8]. In order to protect them from potential related risks of health impairment and sickness absence, which also, from a macro level perspective, negatively affect the educational system due to the increasing staff shortage, interventions are required that aim at optimizing teachers’ job demands and contextual resources or at teaching them strategies for coping with work-related stress.

## Data Availability

The dataset (accession no. ZA7574) used for this article is available as a scientific-use-file (SUF) and can be requested at „BIBB – Federal Institute for Vocational Education and Training “(P.O. Box 20 12 64; 53142 Bonn; Germany; fax number: + 49 – (0)228–107 – 2020). Application form can be downloaded from this website: https://www.bibb.de/de/1403.php. The dataset will be available as ftp-download after approved application.

## Abbreviations

OR: Odds ratio
CI: Confidence interval
